# Phasevarion Mediated Epigenetic Gene Regulation in *Helicobacter pylori*

**DOI:** 10.1371/journal.pone.0027569

**Published:** 2011-12-05

**Authors:** Yogitha N. Srikhanta, Rebecca J. Gorrell, Jason A. Steen, Jayde A. Gawthorne, Terry Kwok, Sean M. Grimmond, Roy M. Robins-Browne, Michael P. Jennings

**Affiliations:** 1 Institute for Glycomics, Griffith University, Gold Coast, Queensland, Australia; 2 Department of Microbiology and Immunology, University of Melbourne, Melbourne, Victoria, Australia; 3 Murdoch Childrens Research Institute, Royal Children's Hospital, Melbourne, Victoria, Australia; 4 Department of Biochemistry and Molecular Biology, Monash University, Melbourne, Victoria, Australia; 5 Institute for Molecular Bioscience, The University of Queensland, Brisbane, Queensland, Australia; Aarhus University, Denmark

## Abstract

Many host-adapted bacterial pathogens contain DNA methyltransferases (*mod* genes) that are subject to phase-variable expression (high-frequency reversible ON/OFF switching of gene expression). In *Haemophilus influenzae* and pathogenic *Neisseria*, the random switching of the *modA* gene, associated with a phase-variable type III restriction modification (R-M) system, controls expression of a phase-variable regulon of genes (a “phasevarion”), via differential methylation of the genome in the *modA* ON and OFF states. Phase-variable type III R-M systems are also found in *Helicobacter pylori*, suggesting that phasevarions may also exist in this key human pathogen. Phylogenetic studies on the phase-variable type III *modH* gene revealed that there are 17 distinct alleles in *H. pylori*, which differ only in their DNA recognition domain. One of the most commonly found alleles was *modH5* (16% of isolates). Microarray analysis comparing the wild-type P12*modH*5 ON strain to a P12Δ*modH*5 mutant revealed that six genes were either up- or down-regulated, and some were virulence-associated. These included *flaA*, which encodes a flagella protein important in motility and *hopG*, an outer membrane protein essential for colonization and associated with gastric cancer. This study provides the first evidence of this epigenetic mechanism of gene expression in *H. pylori*. Characterisation of *H. pylori modH* phasevarions to define stable immunological targets will be essential for vaccine development and may also contribute to understanding *H. pylori* pathogenesis.

## Introduction

The host-adapted pathogen *Helicobacter pylori* is the most common cause of bacterial infection worldwide [1], [2] and is an important etiologic agent of gastritis, peptic ulcers, and gastric cancer [3], [4]. Unless treated, colonization usually persists for life, indicating that *H. pylori* is well adapted to the gastric environment.

In order to adapt its physiology to its environment and ensure survival, *H. pylori* has evolved molecular mechanisms for generating genetic variation [5]. One mechanism is phase-variation, which is the high frequency reversible on/off switching of gene expression. Phase-variation is commonly mediated by mutations in simple tandem DNA repeats in the open reading frame or promoter region of genes encoding surface expressed virulence determinants [6]. The independent, random switching of these genes results in phenotypically diverse populations that can rapidly adapt to host environments and evasion of immune responses [7]. While phase-variation is typically associated with genes encoding surface structures, several host-adapted bacterial pathogens, including *H. pylori*, have DNA methyltransferases (*mod* genes) associated with type III restriction modification (R-M) systems that contain simple tandem DNA repeats which have been proven to phase vary [5], [8], [9].

R-M systems are ubiquitous in bacteria and confer protection to the bacterial host against invasion by foreign DNA [10]. R-M systems are classified into three groups: Types I, II or III on the basis of their subunit composition, DNA cleavage position, sequence-specificity and co-factor requirements [11]. Type III systems are composed of a methyltransferase (modification, *mod*) gene and an endonuclease (restriction, *res*) gene, whose products form a two-subunit enzyme – Mod and Res [12]. In Type III systems Res must form a complex with Mod to be functional [13], although, Mod can function independently of Res [14]. The Mod subunit contains several conserved motifs in the N- and C-terminal regions and the central region contains the DNA-recognition domain that dictates sequence specificity [15].

We have recently shown that in three human pathogens (*Haemophilus influenzae* [16], *Neisseria gonorrhoeae* and *Neisseria meningitidis* [17]) the random switching of the *modA* gene controls expression of a phase-variable regulon of genes (a “phasevarion”), via differential methylation of the genome in the *modA* ON and OFF states. In this study we investigated the *modH* gene, a phase-variable DNA methytransferase of *H. pylori*, to determine if it plays a role in gene regulation.

## Results

### Seventeen *modH* alleles present in *H. pylori*

**Figure 1:**
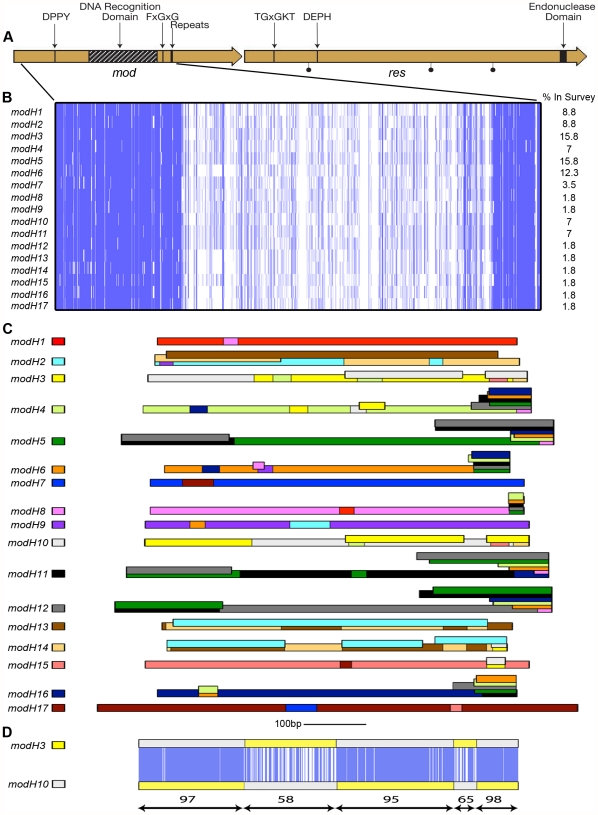
Sequence analysis of the 17 *H. pylori modH* alleles. (A) Diagrammatic representation of the *mod* and *res* genes of *H. pylori*. The methyltransferase gene (*mod*) and restriction endonuclease (*res*) genes and the repeat region that mediates phase-variation are indicated. Type III R-M system conserved motifs are also shown: in *mod*, the catalytic region (DPPY), the AdoMet binding pocket (FxGxG), and the DNA recognition domain (DRD); in *res*, the ATP binding motif (TGxGKT), and ATP hydrolysis motif (DEPH) and the endonuclease domain. A black circle indicates the position of a nonsense mutation or frame-shift mutation in *res*. (B) The variable regions for each of the 17 *modH* alleles in the multiple sequence alignment were aligned in ClustalW and visualised with JalView using the overlay feature. The nucleotides are represented as vertical bars colored according to consensus identity (dark blue >90% identity; light blue >50% identity; white <50% identity or gap). The *modH* alleles were from the following *H. pylori* strains (listed in Table 1); *modH*1 BH13, *modH2* 1061, *modH3* 11637, *modH4* 1134, *modH5* 2A, *modH6* 3A, *modH7* CHP7, *modH8* CHP2, *modH9* CHP4, *modH10* 219, *modH11* GN760, *modH12* L252, *modH13* L264, *modH14* SouthAfrica7, *modH15* Gambia 94/24, *modH16* Cuz20 and *modH17* 908. To generate the amino acid sequences of the DRD region for this comparison the *modH* genes were translated, starting and ending with the residues corresponding to amino acid residues 214 and 449, respectively, with reference to the sequence of the *H. pylori* P12 type III methyltransferase (gb ACJ08645.1). (C) The 17 *modH* alleles are shown as coloured lines. BLASTn matches longer than 20 nucleotides and >90% identity between the 17 *modH* alleles were mapped as a box onto the corresponding allele in the appropriate colour. Detailed information on each individual coloured box is provided in Figure S1 and Table S1. (D) Diagrammatic representation of the tBLASTn match between *modH3* and *modH10*. The nucleotides are represented as vertical bars (dark blue >90% identity; light blue >50% identity; white <50% identity or gap). The numbers below the figure indicate the percent identity as defined by BLASTn for the area between the double-headed arrows.

To investigate if the *modH* gene associated with a type III R-M system of *H. pylori* behaves as a phasevarion [16], [17], we first carried out phylogenetic analysis of *modH*. We have previously reported that *Helicobacter* strains, like the pathogenic *Neisseria*, contain multiple phase-variable type III R-M systems [9]. We defined one of these phase-variable type III R-M systems as *modH*. As with *H. influenzae* and the pathogenic *Neisseria* [16], [17], for each *mod* gene of *H. pylori* there are distinct alleles that differ only in their DNA recognition domain ([15]; see Figure 1A). Comparison of the fully sequenced and annotated *H. pylori* genomes available at the time revealed that there were four distinct alleles of *modH* based on differences in their DNA recognition domain [9]. The *modH* gene contains tracts of simple tandem guanosine repeats that mediate phase-variation of *mod* gene expression.

To investigate whether additional alleles of *modH* are present in *H. pylori*, and to examine the distribution of *modH* alleles and their repeat sequence type and number, sequence analysis of a genetically diverse set of *H. pylori* clinical isolates was performed. This analysis revealed that all strains examined contained the *modH* gene, with thirteen additional *modH* alleles observed, bringing to seventeen the total number of *modH* alleles observed (Table 1, Figure 1B). Here we define *modH* alleles of the same group as sharing more than 90% amino acid identity with other group members in a global pairwise alignment. In previous studies of *mod* genes inter-allelic diversity in the DNA recognition domain is very high with less than 30% amino acid identity shared by DNA recognition domain regions of different groups [16], [17]. In contrast in comparison of the *modH* alleles, there are two groups of alleles (*modH5/modH11/modH12* and *modH3/modH10*) that share large regions of high identity, separated by regions with little or no identity, a pattern suggestive of recombination. The most frequently occurring *modH* alleles observed in the strain survey are *modH3* (16% of isolates tested) and *modH5* (16% of isolates tested). Sequencing of the repeat region of the *modH* alleles revealed that the repeat numbers vary in length between 9 and 15 bp in different strains, resulting in the *mod* genes being in-frame (ON) or out-of-frame (OFF) for expression; consistent with phase-variation of the *modH* gene in this collection of strains (Table 1). Sequence analysis of the corresponding *res* genes in the strain collection revealed that strains F32 (*modH2*), and 52 (*modH11*) have nonsense mutations resulting in truncation of the *res* gene, while strain 51 (*modH3*) has a base pair missing resulting in a frame-shift mutation (Figure 1A, Table 1). Furthermore, for strains 2047 (*modH2*), L101 (*modH3*) and GN760 (*modH11*) a full-length *res* gene could not be amplified (Table 1).

**Table 1:** *ModH* allele and repeat numbers for *H. pylori* clinical isolates and genome sequence strains.

Strain	*modH allele* b	G tract repeat number^a^	Origin	Sequence source(Accession number)
L203	*modH1*	13 (ON)	The Netherlands	This study (HQ734252)
BH13	*modH1*	12 (OFF)	Brazil	This study (HQ734242)
35A	*modH1*	8(OFF)	Norway	CP002096.1 (HMPREF4655_20115)
PeCan4	*modH1*	9 (OFF)	Cancer Patient	CP002074.1 (HPPC_07455)
J99	*modH1*	11 (OFF)	USA	NC_000921.1 (jhp1411)
2047^d^	*modH2*	12 (OFF)	The Netherlands	This study (HQ734240)
1061	*modH2*	12 (OFF)	Canada	This study (HQ734238)
163(A)	*modH2*	11 (OFF)	Brazil	This study (HQ734234)
CHP1	*modH2*	13 (ON)	Australia	This study (HQ734243)
26695	*modH2*	12 (OFF)	UK	NC_000915.1 (HP1522)
2022	*modH3*	15 (OFF)	The Netherlands	This study (HQ734239)
L101^d^	*modH3*	9 (OFF)	The Netherlands	This study (HQ830157)
11637	*modH3*	11 (OFF)	Australia	This study (HQ734241)
L71	*modH3*	10 (ON)	The Netherlands	This study (HQ734251)
SS1	*modH3*	12 (OFF)	Mouse-adapted strain	This study (HQ830158)
F30	*modH3*	14 (OFF)	East Asia	BAJ57491.1 (HPF30_1394)
51^c^	*modH3*	14 (OFF)	Korea	CP000012.1 (KHP_1374)
83	*modH3*	12 (OFF)	USA	CP002605.1 (HMPREF0462_1519)
HPAG_1	*modH3*	13 (ON)	Sweden	CP000241.1 (HPAG1_1393)
1134	*modH4*	14 (OFF)	Canada	This study (HQ734257)
Sat464	*modH4*	10 (ON)	Peru	CP00207.1 (HPSAT_07320)
98-10	*modH4*	11 (OFF)	Japan	ABSX01000015.1 (HP9810_885g17)
HPG27	*modH4*	11 (OFF)	Italy	CP001173.1 (HPG27_1444)
2025	*modH5*	15 (OFF)	The Netherlands	This study (JN974761)
CHP5	*modH5*	12 (OFF)	Australia	This study (HQ734246)
KC7617	*modH5*	11 (OFF)	Canada	This study (HQ734250)
5A	*modH5*	10 (ON)	The Netherlands	This study (HQ734233)
P12	*modH5*	10 (ON)	Germany	CP001217.1 (HPP12_1497)
2A	*modH5*	9 (OFF)	The Netherlands	This study (HQ734231)
HPB8	*modH5*	13 (ON)	Gerbil-adapted strain	NC_014257 (HPB8_7)
India7	*modH5*	14 (OFF)	India	CP002331.1 (HPIN_07505)
L251	*modH5*	9 (OFF)	The Netherlands	This study (HQ734253)
3A	*modH6*	12 (OFF)	The Netherlands	This study (HQ734232)
L2624	*modH6*	12 (OFF)	The Netherlands	This study (HQ734256)
Shi470	*modH6*	10 (ON)	Asia/South America	CP001072.2 (HPSH_07815)
F32^c^	*modH6*	12 (OFF)	East Asia	BAJ58990.1 (HPF32_1408)
F57	*modH6*	11 (OFF)	East Asia	BAJ60509.1 (HPF57_1435)
Lithuania75	*modH6*	12 (OFF)	Lithuania	CP002334.1 (HPLT_07575)
758TM	*modH6*	9 (OFF)	Canada	This study (HQ734236)
CHP7	*modH7*	15 (OFF)	Australia	This study (HQ734248)
AS620	*modH7*	10 (ON)	Canada	This study (JN974762)
CHP2	*modH8*	11 (OFF)	Australia	This study (HQ734244)
CHP4	*modH9*	13 (ON)	Australia	This study (HQ734245)
L80	*modH10*	11 (OFF)	The Netherlands	This study (JN974763)
219	*modH10*	9 (OFF)	Brazil	This study (HQ734235)
SJM180	*modH10*	9 (OFF)	Peru	NC_014560.1 (HPSJM_07770)
CHP6	*modH10*	10 (ON)	Australia	This study (HQ734247)
GN760^d^	*modH11*	11 (OFF)	Canada	This study (HQ734237)
F16	*modH11*	12 (OFF)	East Asia	AP011940.1 (HPF16_1417)
52^c^	*modH11*	11 (OFF)	Korea	CP001680.1 (HPKB_1423)
CHP8	*modH11*	10 (ON)	Australia	This study (HQ734249)
L252	*modH12*	10 (ON)	The Netherlands	This study (HQ734254)
L264	*modH13*	13 (OFF)	The Netherlands	This study (HQ734255)
SouthAfrica7	*modH14*	9 (OFF)	South Africa	CP002336.1 (HPSA_07265)
Gambia94/24	*modH15*	9 (OFF)	Gambia	CP002332.1 (HPGAM_08025)
Cuz20	*modH16*	11 (OFF)	Peru	CP002076.1 (HPCU_07650)
908	*modH17*	12 (OFF)	Africa	CP002184.1 (hp908_1508)

a Number and expression state of poly-guanosine repeats within the *mod* gene; in-frame (ON) or out-of-frame (OFF).

b A strain was defined as having the *modH* allele if the DNA recognition region was ≥90% identical at the nucleotide level to the *modH* gene of *H. pylori*. A strain was defined as having a particular *modH* allele if the DNA recognition region was ≥90% identical at the amino acid and nucleotide level to the *modH* allele. Refer to Figure 1.

c *res* gene contains a nonsense mutation (strain F32, nucleotide 1587 and strain 52, nucleotide 537 change from G to T) or missing base pair resulting in a frame-shift mutation (strain 51, nucleotide 2054).

d *res* gene not detected.

Representatives of all 17 *modH* alleles were compared by multiple sequence alignment. Figure 1B illustrates the diversity seen throughout the DNA recognition domain of the *modH* alleles at the nucleotide level. There are several short regions of high similarity within the DNA recognition domain. The mosaic pattern observed in the alignment schematic (Figure 1B) suggests that large segments have been deleted or replaced via genetic recombination. To examine recombination within the DNA recognition domains in more detail and to determine its contribution to *modH* allele diversity, we undertook all versus all BLASTn searches using the 13 representative *modH* sequences (Figure 1C). The number of reciprocal exchanges identified gave a clear indication that the *modH* alleles have recombined in the past. By using this approach we identified new relationships between the *modH* alleles. Some alleles were found to have undergone recombination more readily than others to generate new alleles. For example, *modH5*, *modH11* and *modH12* share near identical 5′ and 3′ regions, but each has a different central fragment suggesting recent origin from an ancestral allele (Figure 1C). Closer examination of these regions reveals that the 5′ end of each allele (*modH5*, *modH11* and *modH12*) matches the first 200 nucleotides of the other two alleles. For example, *modH5* has matches to nucleotides 1–177 of *modH11* and nucleotides 1–186 of *modH12*, *modH11* has matches to *modH5* (1–186) and *modH12* (1–172) and *modH12* has matches to *modH5* (1–177) and *modH11* (1–172) (Figure S1, Table S1). Similarly the 3′ end of *modH5* has matches to regions related to *modH11* (nucleotides 517–711) and *modH12* (nucleotides 517–711) as well as *modH4* (nucleotides 641–711), *modH*6 (nucleotides 647–711), *modH*8 (nucleotides 688–711) and *modH*16 (nucleotides 641–711) (Figure S1, Table S1). These regions of similarity are also observed in the 3′ ends of *modH11* and *modH12* (Figure S1, Table S1). Likewise, *modH3* and *modH10* also appear to have originated from a single allele that has undergone at least two recombination events to generate the diversity that now distinguishes them from one another (Figure 1D).

### Analysis of differentially expressed genes in the *H. pylori modH5* phasevarion

To determine whether phase-variation of the *modH* allele in *H. pylori* resulted in changes in global gene expression, we conducted a study using *H. pylori* strain P12, which expresses the most common *modH5* allele. The *modH5* gene was inactivated by insertion of a *cat* cassette to make the mutant strain P12Δ*modH5*. Wild-type P12*modH5* ON and P12Δ*modH5* were compared by microarray analysis using *H. pylori* P12 genome arrays (Methods). Six genes were found to be differentially expressed by a ratio of 1.6-fold or more, with 2 genes up-regulated in P12Δ*modH5* relative to wild-type and 4 genes down-regulated. These data confirmed that *modH5* phase-variation has an influence on gene expression (Table 2). One gene with an increased expression of 2.4 fold in the *modH* mutant encodes the surface exposed protein, HopG (also known as HopY), a potential vaccine candidate [18]. HopG is required for colonization [19] and may be associated with gastric cancer [20]. Two genes associated with flagella showed increased expression in the *modH* ON strain. FlaA is the major component of the flagellar filament [21], [22] and is required for normal motility, which is essential for colonization [23] and the establishment of persistent infection [24]. HPP12_904 is homologous to the flagellar hook-length control protein FliK that is also essential for motility [25].

**Table 2:** Differentially expressed genes in *H. pylori* wild-type P12 *modH5* ON versus the mutant strain P12Δ*modH5*.

Gene ID^a^	Gene Name	Ratio^b^	B-Stat^c^	QRT-PCR^d^
**Reduced expression in the ** ***H. pylori*** ** strain P12 ** ***modH5*** ** mutant**				
HPP12_1497	type III R-M system methyltransferase	−5.94	6.350	
HPP12_0609	flagellin A	−1.97	3.583	−2.00 ± 0.609
HPP12_0904	hypothetical protein	−1.79	0.831	−1.92 ± 0.615
HPP12_0870	flagellar hook protein Flg	−1.57	0.118	
**Increased expression in ** ***H. pylori*** ** strain P12 ** ***modH5*** ** mutant**				
HPP12_0255	hypothetical protein	1.80	0.667	4.72 ± 0.686
HPP12_0253	outer membrane protein HopG	2.38	1.631	3.12 ± 0.427

a The genes listed are either down- or up- regulated in the *H. pylori* P12Δ*modH5* mutant strain. The identity of the gene is indicated with the gene ID in the annotation of the *H. pylori* P12 genome [42].

b The ratio presented is the mean of *H. pylori* P12Δ*modH5* mutant:wild-type P12*modH5* ON from multiple replicate spots on three independent microarrays. Only those genes with an expression ratio ≥1.5-fold were included in this study.

c Determined using LIMMA [41].

d Gene expression confirmed by quantitative RT-PCR (QRT-PCR) in the wild-type *H. pylori* P12*modH5* ON strain and the *H. pylori* P12*modH5* OFF strain. Results for each gene were as follows: HPP12_253 (5.10± 0.375), HPP12_255 (3.50± 0.346), HPP12_609 (−3.81 ± 0.184), HPP12_904 (−3.52± 0.290).

Quantitative real time PCR (QRT-PCR) confirmed that *flaA* and HPP12_904 were expressed at a higher level in the wild-type P12*modH5* ON parent strain compared to P12Δ*modH5*, while *hopG* and HPP12_0255 were expressed at a higher level in the P12Δ*modH5* mutant compared to P12*modH5* ON (Table 2). QRT-PCR that compared the wild-type P12*modH5* ON (G_10_) strain to a P12*modH5* OFF strain (G_6_TG_4_), also confirmed the microarray results (Table 2).

## Discussion

We recently confirmed gene regulation as a function of phase-variable type III R-M systems in the human pathogens *H. influenzae* [16] and pathogenic *Neisseria* [17], thus defining a new paradigm in bacterial gene regulation “the phase-variable regulon; “phasevarion” [9]. In this study we investigated whether the phase-variable type III R-M systems described in *H. pylori* serve a similar regulatory function.

*H. pylori* contain a number of phase-variable type II and type III R-M systems [9], [26], [27], as well as several active orphan type methyltransferases [28], [29], [30], suggesting that these methyltransferases may have functions other than restriction, such as gene regulation [31]. Additionally, *H. pylori* strains have been found to have inactive type III and II *res* genes, indicating that DNA restriction may not be the function of phase-variable R-M systems [8], [31]. Recently, methylation by a putative phase-variable *mod* gene associated with a type II R-M system in *H. pylori* (M.HpyAIV) was shown to influence gene expression of *katA* [31]. The M.HpyAIV gene has also been associated with the induction of a more robust host response in mice, suggesting an involvement in gene regulation [32].

In type III R-M systems DNA sequence specificity is conferred by the Mod subunit [15]. Differences in the *modA* DNA recognition domain have previously been observed in *H. influenzae* with 17 distinct *modA* alleles defined in this organism [33], [34]. In pathogenic *Neisseria*, we identified three distinct *modA* alleles and two distinct *modB* alleles [17]. Our phylogenetic studies on the *modH* gene of a collection of *H. pylori* clinical isolates revealed that there are 17 distinct *modH* alleles based on differences in their DNA recognition domain. In pathogenic *Neisseria*, strains with the same DNA recognition domain regulated the same set of genes, while those with different *mod* alleles regulate the expression of different sets of genes [17]. Seventeen distinct *modH* alleles in *H. pylori* suggest that 17 distinct phasevarions exist. *ModH3* and *modH5* were the most frequent alleles observed in clinical isolates.

Further studies on the *modH5* phasevarion were conducted using microarray to compare *H. pylori* strain P12, which expresses the *modH5* allele, to a mutant strain. Of the genes regulated by the *modH5* phasevarion, two encode proteins that have important roles in motility, FlaA and FliK. Motility is an essential factor for the colonization and persistence of *H. pylori* in the human stomach [24] and therefore flagella have an important role in virulence. In addition, *H. pylori* FlaA has low intrinsic capacity to activate innate immunity via the Toll-like receptor 5 [35], [36], [37]. Therefore, altered expression of flagella may be advantageous for the adaptation of *H. pylori* to alternate host environments and in evading the host immune response. The gene encoding the essential outer membrane protein for colonization, HopG [19] was also found to be regulated by the *modH5* phasevarion. Bacterial adherence mediated by HopG and outer membrane proteins is thought to play an important role in the colonization of the gastric epithelium by *H. pylori* [38], making HopG an attractive vaccine target [18]. Hence phasevarion mediated phase-variation of *hopG* has the potential to mediate escape from the host immune response.

Only a relatively small number of genes were found to be under the control of the *modH5* phasevarion. This may be the full extent of the regulon, or only be a sub-set of the regulon due to the analysis being done under standard in vitro culture conditions. Differences in gene expression can only be detected if the genes in question are being expressed. Using different physiologically relevant conditions, such as specific pH conditions that reflect the gastric environment, may result in more genes being found to be under the influence of the *modH5* phasevarion.

Here we provide evidence for a role for phase-variable *mod* genes associated with type III R-M systems in gene regulation in *H. pylori*. Although we cannot exclude another as yet undescribed role for these *modH* phase variation in *H. pylori* biology, we have confirmed phasevarion mediated epigenetic mechanism of gene expression does operate in *H. pylori*. Further characterisation of this phasevarion will contribute to an improved understanding of *H. pylori* pathogenesis and may guide vaccine development for this important human pathogen by defining stably expressed immunological targets in *modH5* strains. Based on our previous studies [9] it is likely that other *H. pylori modH* alleles also function as phasevarions and regulate gene expression. Future studies in *H. pylori* involving gene regulation, host/pathogen interactions or vaccine development need to control for the potential for *modH* phase variation to alter global gene expression.

## Materials and Methods

### Bacterial strains and growth conditions

*H. pylori* strains were routinely grown from glycerol stocks for 2 days on GC agar (Oxoid, Basingstoke, UK) plates supplemented with 10% (v/v) horse serum (Invitrogen Corp, Carlsbad, CA), vitamin mix and antibiotics (nystatin, 20 mg/ml; trimethoprim, 2.5 mg/ml; vancomycin, 10 mg/ml) in a microaerobic atmosphere as described previously [39]. Plates for cultivation of mutant strains were further supplemented with chloramphenicol (4 mg/ml for routine culture, 10 mg/ml for selection of transformants).

### DNA manipulation and analysis

All enzymes were sourced from New England Biolabs. Sequencing was performed on PCR products using QiaQuick PCR purification kit (Qiagen) and Big-Dye (Perkin Elmer) sequencing kits. Data were analysed using MacVector v11.0 (Accelrys).

ModH alleles (formerly called ModC [9]) were classified as ModH1-13 according to all-versus-all global pairwise amino acid alignments of the ModH DRD region and a within-group minimum identity cut-off of 90%. Global pairwise alignments were calculated with a dynamic programming technique as implemented in Jalview (http://www.jalview.org/) (alignment parameters: BLOSUM62 substitution matrix, gap-open penalty of 12, gap-extend penalty of 2). Inter-allele comparisons were carried out using all versus all BLASTn and BLASTp comparisons of representative ModH DRD alleles using stand-alone NCBI BLAST without filters (version 2.2.18). Amino acid and nucleotide sequences were aligned using ClustalX (version 2.0.11). Multiple alignments were viewed and edited in Jalview (42). The GenBank accession numbers are HQ734231–HQ734257, HQ830157–HQ830158 and JN974761–JN974763.

### *mod* and *res* specific PCR

The *modH* gene and *resH* gene were amplified and sequenced using the primers listed in Table 3. *H. pylori* clinical isolates were used as templates (Table 1). The reaction was performed in 50 µl using KOD (Novagen) reagents_,_ and 1 unit of KOD DNA polymerase with the following cycling conditions for the *modH* gene: 30 cycles of 94°C for 30 sec, 50°C for 30 sec, 70°C for 1 min and 1 cycle of 72°C for 5 min with 5 µM of the primer pair HP_MODHF1 and HP_MODHR5. A 693 bp region containing the DNA recognition domain (603 bp downstream of HP_MODHF1 and 640 bp upstream from HP_MODHR5) was compared to the *H. pylori* genome strains to determine the *modH* allele (Table 1). The primers HP_MODHREPEATF and HP_MODHR4 were used to sequence the repeat region. The *resH* gene was amplified using the primer pair HP_RESHF1 and HP_RESHR1 with the following cycling conditions: 30 cycles of 94°C for 30 sec, 50°C for 30 sec, 72°C for 2 min and 1 cycle of 72°C for 5 min. Only the regions containing the conserved motifs and nonsense mutations were sequenced using the primers HP_RESHF1, HP_RESHR1 and HP_RESHR7. PCR products were cleaned using the QIAquick PCR Purification Kit (Qiagen).

**Table 3:** Primers used for *modH* allele study.

Primer	Nucleotide Sequence 5′-3′
HP_MODHF1	GGATAGAGATGCAAAATAAAGAAATTG
HP_ MODHF2	CTCATCAAGGGCGATAATTTAGACG
HP_ MODHF3	CCAATGAAGAGGTTTTAAAAAC
HP_MODHF4	ACTCAAACTTTTATGCGATG
HP_MODHF5	GAGAGTAATAAGAGCGATTATC
HP_MODHF6	GGCGCTTCATTCTCGTCCAG
HP_MODHREPEATF	GCCGGGAGCGGGACAACCGCGCAT
HP_MODHR1	GTTTTTAAAACCTCTTCATTGG
HP_MODHR2	CCGTCTTGTTTGAGCAAATCTTTAG
HP_MODHR3	GATAATCGCTCTTATTACTCTC
HP_ MODHR4	CTGGACGAGAATGAAGCGCC
HP_MODHR5	CTACCCCCTAATCTTTAAATCGCC
HP_RESHF1	GGCGATTTAAAGATTAGGGGGTAG
HP_RESHR1	GTTCCATGTGAAACATTAGAG
HP_RESHR7	CTTTTTTATGCGTCGTAACCGAAAC
HP_0253F	CTGGCACGGACTTTTTATG
HP_0253R	CCCAAGTGTTACCCGCTAT
HP12_0255F	GCGCTCTAAGAATGGAGATAGAATATTAC
HP12_0255R	GCTAGAATATAATCTTTTTCTAAAACTTTTAAATCC
HP12_0609F	TAGTTCAGCAGGCACAGGGATTGG
HP12_0609R	TGGTGATAACGCTCGCATAAGC
HP12_0904F	AACGCTAAAGAGCCAAAAACCC
HP12_0904R	GAGTTGTGGTCGCTTGAATGTTG
16SF	ACGGAGGGTGCGAGCGTTAATC
16SR	TCGCCTTCGCAATGAGTATTCCT
RGRM4F	ATGCAAAATAAAGAAATTGGTG
RGRM4R	CTACCCCCTAATCTTTAAATCGCC
RGRM4Fmut	CGGATCCGTGGGGGATATAGAAATGAG
RGRM4Fmut1	CGGATCCTAAATTATCGCCCTTGATGAG
RGRM4Fmut2	CCGGATCCGGTGAAGCCCATCAAAAGGATTTG
RGRM4Rmut3	CCGGATCCTTAAATAACCCCTCCCCCCTC

Underlined sequences represent introduced BamHI restriction sites.

### Construction of knockout mutant and OFF mutant of the *modH5* gene of *H. pylori* strain P12

Two mutants of *modH* were made using strain *H. pylori* P12. In strain P12 the *modH5 gene* contains a G_10_ tract in the coding sequence of the gene and is in-frame for expression of a full-length *modH* gene; hence it is defined as “ON”. The complete ORF of P12*modH*5 was amplified using Vent DNA polymerase and primer pair RGRM4F/RGRMR4 and A-tailed before cloning into pGEM-T easy (Promega). The first mutant, P12Δ*modH*5, was produced by allelic exchange of a 1480 bp region of *modH* containing the DNA binding region and poly-G tract, with a choloramphenicol resistance cassette (*cat*) lacking a transcriptional terminator [39]. The cloned ORF was inversely amplified using primer pair RGRM4Fmut/RGRM4Rmut1 to excise the 1480 bp region and introduce BamHI sites, which were used to ligate BamHI-cut *cat*. The second mutant, P12*modH*5 OFF, carried an insertion in the polyG tract to alter it to N_11_, resulting in a frame-shift mutation to an “OFF” phase. The cloned ORF was inversely amplified using primer pair RGRMFmut2/RGRM4Rmut3 to insert a silent thymidine residue into the poly-G tract, changing G_10_, to 5′-GGGGGGTGGGG-3′, excising a 270 bp region, and introducing BamHI sites for ligation with BamHI-cut *cat*. Both mutagenesis constructs carried the *cat* cassette in the same orientation as *modH*. For transformation of *H. pylori* P12, the linear mutagenesis cassettes were amplified using primer pair RGRM4F/RGRM4R. Naturally competent P12 was transformed using the purified PCR product as described previously [39] and transformants were screened by sequence analysis.

### RNA extraction

Triplicate cultures of *H. pylori* strain wild-type P12*modH5* ON, P12*modH5* OFF and the P12Δ*modH5* mutant, were grown to exponential phase (optical density at 550 nm  =  0.9) in BHI broth (Oxoid) supplemented with 10% (v/v) fetal bovine serum (Invitrogen), vitamin mix and vancomycin. Bacterial cells were stabilized using RNAprotect Bacteria Reagent (Qiagen) prior to RNA extraction and approximately 50 µg of total RNA was prepared from each sample using the RNeasy Maxi Kit according to the manufacturer's instructions (Qiagen). The integrity and concentration of RNA were determined via micro-fluidic analysis on a bio-analyser (Agilent Technologies).

### Microarray analysis

Custom Agilent 8×15 k oligonucleotide microarrays (Agilent, CA, USA) were designed based on the publically available sequence of *H. pylori* P12 (NC_011498) using E-array (Agilent, CA, USA). Reverse transcription reactions were performed in 40 µl volumes, containing 10 µg total RNA, 300 ng random hexamers, 0.5 mM dNTPs and 300 U SuperScript III Reverse Transcriptase (Invitrogen) at 42°C for 2.5 h. RNA contamination was removed from the cDNA by the addition of NaOH followed by column purification (Qiagen minElute, Qiagen). A total of 1 µg of purified cDNA was labeled using KREAtech Cy3-ULS (KREAtech, The Netherlands), and 625 ng was used to hybridize Agilent 8×15 k microarrays as per the manufacturer's instructions.

Hybridized arrays were scanned on an Agilent Genepix G2565BA scanner, and features were extracted using Feature Extraction V9.5 (Agilent, CA, USA). Analysis was performed using LIMMA [40] as follows. Background correction was applied, spots from duplicate probes were averaged and log transformed. Between-array quantile normalization was then applied to the log transformed spot intensities. A moderated t-test on the normalized log intensities was performed to identify differentially expressed genes and the False Discovery Rate (FDR) used to control for multiple testing. Genes were ranked using the B-statistic (B-stat) method where both fold change and variance of signals in replicates are used to determine the likelihood that genes are truly differentially expressed. A threshold in the B- stat of 0.0 was adopted as genes with a B score >0 have a >50% probability of being truly differentially expressed [41]. All experimental data are available online at the NCBI Gene Expression Omnibus (http://www.ncbi.nlm.nih.gov/geo/) submission number GSE26759. All data is MIAME compliant.

### Quantitative Real-Time PCR

Oligonucleotides (Table 3) were designed using Primer Express 1.0 software (ABI Prism; PE Biosystems) and are named according to the open reading frame (ORF) being amplified. All real-time PCR reactions were performed in a 25 µl mixture containing 1 in 5 dilution of cDNA preparation (5 µl), 10× SYBR Green buffer (PE Applied Biosystems) and 5 µM of each primer. We used 16S RNA as the control in each quantitative PCR. Amplification and detection of specific products were performed with the ABI Prism 7700 sequence-detection system (PE Applied Biosystems) with the following cycle profile: 95°C for 10 min, followed by 45 cycles of 95°C for 15 sec and 60°C for 1 min. Data were analyzed with ABI prism 7700 (version 1.7) analysis software. Relative gene expression between the P12Δ*modH5* mutant and wild-type P12*modH5* ON and P12*modH5* OFF and wild-type P12*modH5* ON was determined using the 2^ΔΔCT^ relative quantification method.

## Supporting Information

Figure S1 **Diagrammatical representation of the 17** ***modH*** **alleles of** ***H. pylori*** **.** BLASTn was used to identify reciprocal exchanges between the *modH* DNA recognition domains of the following *H. pylori* strains (listed in Table 1); *modH*1 BH13, *modH2* 1061, *modH3* 11637, *modH4* 1134, *modH5* 2A, *modH6* 3A, *modH7* CHP7, *modH8* CHP2, *modH9* CHP4, *modH10* 219, *modH11* GN760, *modH12* L252, *modH13* L264, *modH14* SouthAfrica7, *modH15* Gambia 94/24, *modH16* Cuz20 and *modH17* 908. Each unique *modH* DNA recognition domain is represented as a coloured box. BLASTn matches longer than 20 nt and >90% identity were mapped on to the corresponding allele in the appropriate colour. The number above the coloured boxes corresponds to Table S1 that contains details of the start and stop positions of each exchange. The nucleotide positions correspond to the DNA recognition domain only.
(DOCX)

Table S1 **Details of matches shown diagrammatically in Figure 1 (coordinates shown in Figure S1).**
(DOCX)
