# Supplementary material for: Phasevarion Mediated Epigenetic Gene Regulation in Helicobacter pylori
Source: PLoS One. 2011 Dec 5;6(12):e27569. doi: 10.1371/journal.pone.0027569 (PMC3230613; doi:10.1371/journal.pone.0027569)
Supplement: Figure S1 — Diagrammatical representation of the 17 modH alleles of H. pylori . BLASTn was used to identify reciprocal exchanges between the modH DNA recognition domains of the following H. pylori strains (listed in Table 1); modH1 BH13, modH2 1061, modH3 11637, modH4 1134, modH5 2A, modH6 3A, modH7 CHP7, modH8 CHP2, modH9 CHP4, modH10 219, modH11 GN760, modH12 L252, modH13 L264, modH14 SouthAfrica7, modH15 Gambia 94/24, modH16 Cuz20 and modH17 908. Each unique modH DNA recognition domain is represented as a coloured box. BLASTn matches longer than 20 nt and >90% identity were mapped on to the corresponding allele in the appropriate colour. The number above the coloured boxes corresponds to Table S1 that contains details of the start and stop positions of each exchange. The nucleotide positions correspond to the DNA recognition domain only. (DOCX) [file pone.0027569.s001.docx]

**Figure S1. Diagrammatical representation of the 17 *modH* alleles of *H. pylori***

BLASTn was used to identify reciprocal exchanges between the *modH* DNA recognition domains of the following *H. pylori* strains (listed in Table 1); *modH*1 BH13, *modH2* 1061, *modH3* 11637, *modH4* 1134, *modH5* 2A, *modH6* 3A, *modH7* CHP7, *modH8* CHP2, *modH9* CHP4, *modH10* 219, *modH11* GN760, *modH12* L252, *modH13* L264, *modH14* SouthAfrica7, *modH15* Gambia 94/24, *modH16* Cuz20 and *modH17* 908. Each unique *modH* DNA recognition domain is represented as a coloured box. BLASTn matches longer than 20 nt and >90% identity were mapped on to the corresponding allele in the appropriate colour. The number above the coloured boxes corresponds to Table S1 that contains details of the start and stop positions of each exchange. The nucleotide positions correspond to the DNA recognition domain only.
