# Supplementary material for: Phasevarion Mediated Epigenetic Gene Regulation in Helicobacter pylori
Source: PLoS One. 2011 Dec 5;6(12):e27569. doi: 10.1371/journal.pone.0027569 (PMC3230613; doi:10.1371/journal.pone.0027569)
Supplement: Table S1 — Details of matches shown diagrammatically in Figure 1 (coordinates shown in Figure S1). (DOCX) [file pone.0027569.s002.docx]

**Table S1. Details of matches shown diagrammatically in Figure 1 (coordinates shown in Figure S1).**

Matches to *modH1* (BH13)

| **Fragment Number^a^** | ***modH* Match^b^** | **Accession^c^** | **5’ Hit^c^** | **3’ Hit^d^** |
| --- | --- | --- | --- | --- |
| 1 | 8 | HQ734244 | 110 | 134 |

Matches to *modH2* (L2624)

| **Fragment Number^a^** | ***modH* Match^b^** | **Accession^c^** | **5’ Hit^c^** | **3’ Hit^d^** |
| --- | --- | --- | --- | --- |
| 2 | 14 | CP002336 | 1 | 195 |
| 3 | 9 | HQ734245 | 8 | 29 |
| 4 | 13 | HQ734255 | 19 | 531 |
| 5 | 14 | CP002336 | 293 | 425 |
| 6 | 14 | CP002336 | 446 | 564 |

Matches to *modH3* (11637)

| **Fragment Number^a^** | ***modH* Match^b^** | **Accession^c^** | **5’ Hit^c^** | **3’ Hit^d^** |
| --- | --- | --- | --- | --- |
| 7 | 10 | HQ734235 | 1 | 175 |
| 8 | 4 | HQ734257 | 206 | 236 |
| 9 | 10 | HQ734235 | 325 | 518 |
| 10 | 4 | HQ734257 | 345 | 386 |
| 11 | 10 | HQ734235 | 556 | 624 |
| 12 | 15 | CP002332 | 562 | 592 |
| 13 | 14 | CP002336 | 601 | 624 |

Matches to *modH4* (1134)

| **Fragment Number^a^** | ***modH* Match^b^** | **Accession^c^** | **5’ Hit^c^** | **3’ Hit^d^** |
| --- | --- | --- | --- | --- |
| 14 | 16 | CP002076 | 78 | 107 |
| 15 | 3 | HQ734245 | 242 | 272 |
| 16 | 10 | HQ734235 | 342 | 368 |
| 17 | 3 | HQ734245 | 357 | 398 |
| 18 | 12 | HQ734254 | 541 | 639 |
| 19 | 11 | HQ734237 | 554 | 639 |
| 20 | 6 | HQ734232 | 568 | 639 |
| 21 | 16 | CP002076 | 570 | 639 |
| 22 | 5 | HQ734231 | 571 | 639 |
| 23 | 8 | HQ734244 | 615 | 639 |

Matches to *modH5* (2A)

| **Fragment Number^a^** | ***modH* Match^b^** | **Accession^c^** | **5’ Hit^c^** | **3’ Hit^d^** |
| --- | --- | --- | --- | --- |
| 24 | 12 | HQ734254 | 1 | 177 |
| 25 | 11 | HQ734237 | 1 | 186 |
| 26 | 12 | HQ734254 | 517 | 711 |
| 27 | 11 | HQ734237 | 517 | 711 |
| 28 | 16 | CP002076 | 641 | 711 |
| 29 | 4 | HQ734257 | 641 | 711 |
| 30 | 6 | HQ734232 | 647 | 711 |
| 31 | 8 | HQ734244 | 688 | 711 |

Matches to *modH6* (1061)

| **Fragment Number^a^** | ***modH* Match^b^** | **Accession^c^** | **5’ Hit^c^** | **3’ Hit^d^** |
| --- | --- | --- | --- | --- |
| 32 | 16 | CP002076 | 66 | 93 |
| 33 | 8 | HQ734244 | 154 | 173 |
| 34 | 9 | HQ734245 | 162 | 188 |
| 35 | 4 | HQ734257 | 526 | 597 |
| 36 | 16 | CP002076 | 532 | 597 |
| 37 | 11 | HQ734237 | 535 | 597 |
| 38 | 12 | HQ734254 | 535 | 597 |
| 39 | 5 | HQ734231 | 535 | 597 |

Matches to *modH7* (CHP7)

| **Fragment Number^a^** | ***modH* Match^b^** | **Accession^c^** | **5’ Hit^c^** | **3’ Hit^d^** |
| --- | --- | --- | --- | --- |
| 40 | 17 | CP002184 | 54 | 105 |

Matches to *modH8* (CHP2)

| **Fragment Number^a^** | ***modH* Match^b^** | **Accession^c^** | **5’ Hit^c^** | **3’ Hit^d^** |
| --- | --- | --- | --- | --- |
| 41 | 1 | HQ734242 | 311 | 335 |
| 42 | 4 | HQ734257 | 588 | 611 |
| 43 | 6 | HQ734232 | 588 | 612 |
| 44 | 11 | HQ734237 | 589 | 611 |
| 45 | 12 | HQ734254 | 589 | 612 |
| 46 | 5 | HQ734231 | 589 | 612 |

Matches to *modH9* (CHP4)

| **Fragment Number^a^** | ***modH* Match^b^** | **Accession^c^** | **5’ Hit^c^** | **3’ Hit^d^** |
| --- | --- | --- | --- | --- |
| 47 | 6 | HQ734232 | 74 | 99 |
| 48 | 2 | HQ734238 | 238 | 304 |

Matches to *modH10* (219)

| **Fragment Number^a^** | ***modH* Match^b^** | **Accession^c^** | **5’ Hit^c^** | **3’ Hit^d^** |
| --- | --- | --- | --- | --- |
| 49 | 3 | HQ734245 | 1 | 175 |
| 50 | 3 | HQ734245 | 328 | 521 |
| 51 | 4 | HQ734257 | 333 | 359 |
| 52 | 3 | HQ734245 | 559 | 627 |
| 53 | 15 | CP002332 | 565 | 595 |
| 54 | 14 | CP002336 | 602 | 627 |

Matches to *modH11* (GN760)

| **Fragment Number^a^** | ***modH* Match^b^** | **Accession^c^** | **5’ Hit^c^** | **3’ Hit^d^** |
| --- | --- | --- | --- | --- |
| 55 | 12 | HQ734254 | 1 | 172 |
| 56 | 5 | HQ734231 | 1 | 186 |
| 57 | 5 | HQ734231 | 368 | 393 |
| 58 | 12 | HQ734254 | 475 | 690 |
| 59 | 5 | HQ734231 | 496 | 690 |
| 60 | 4 | HQ734257 | 608 | 690 |
| 61 | 6 | HQ734232 | 626 | 690 |
| 62 | 16 | CP002076 | 634 | 690 |
| 63 | 8 | HQ734244 | 667 | 690 |

Matches to *modH12* (L252)

| **Fragment Number^a^** | ***modH* Match^b^** | **Accession^c^** | **5’ Hit^c^** | **3’ Hit^d^** |
| --- | --- | --- | --- | --- |
| 64 | 11 | HQ734237 | 1 | 172 |
| 65 | 5 | HQ734231 | 1 | 177 |
| 66 | 11 | HQ734237 | 501 | 717 |
| 67 | 5 | HQ734231 | 523 | 717 |
| 68 | 16 | CP002076 | 616 | 717 |
| 69 | 4 | HQ734257 | 622 | 717 |
| 70 | 6 | HQ734232 | 653 | 717 |
| 71 | 8 | HQ734244 | 694 | 717 |

Matches to *modH13* (L264)

| **Fragment Number^a^** | ***modH* Match^b^** | **Accession^c^** | **5’ Hit^c^** | **3’ Hit^d^** |
| --- | --- | --- | --- | --- |
| 72 | 14 | CP002336 | 7 | 531 |
| 73 | 2 | HQ734238 | 19 | 531 |
| 74 | 14 | CP002336 | 293 | 459 |
| 75 | 14 | CP002336 | 498 | 531 |

Matches to *modH14* (SouthAfrica7)

| **Fragment Number^a^** | ***modH* Match^b^** | **Accession^c^** | **5’ Hit^c^** | **3’ Hit^d^** |
| --- | --- | --- | --- | --- |
| 76 | 2 | HQ734238 | 1 | 195 |
| 77 | 13 | HQ734255 | 7 | 195 |
| 78 | 2 | HQ734238 | 290 | 422 |
| 79 | 13 | HQ734255 | 290 | 456 |
| 80 | 2 | HQ734238 | 443 | 561 |
| 81 | 13 | HQ734255 | 495 | 528 |
| 82 | 10 | HQ734235 | 536 | 561 |
| 83 | 3 | HQ734245 | 538 | 561 |

Matches to *modH15* (Gambia 94/24)

| **Fragment Number^a^** | ***modH* Match^b^** | **Accession^c^** | **5’ Hit^c^** | **3’ Hit^d^** |
| --- | --- | --- | --- | --- |
| 84 | 17 | CP002184 | 321 | 340 |
| 85 | 10 | HQ734235 | 562 | 592 |
| 86 | 3 | HQ734245 | 562 | 592 |

Matches to *modH16* (Cuz20)

| **Fragment Number^a^** | ***modH* Match^b^** | **Accession^c^** | **5’ Hit^c^** | **3’ Hit^d^** |
| --- | --- | --- | --- | --- |
| 87 | 6 | HQ734232 | 69 | 96 |
| 88 | 4 | HQ734257 | 69 | 100 |
| 89 | 12 | HQ734254 | 487 | 591 |
| 90 | 4 | HQ734257 | 522 | 591 |
| 91 | 5 | HQ734231 | 523 | 591 |
| 92 | 6 | HQ734232 | 526 | 591 |
| 93 | 11 | HQ734237 | 535 | 591 |

Matches to *modH17* (908)

| **Fragment Number^a^** | ***modH* Match^b^** | **Accession^c^** | **5’ Hit^c^** | **3’ Hit^d^** |
| --- | --- | --- | --- | --- |
| 94 | 7 | HQ734248 | 309 | 360 |
| 95 | 15 | CP002332 | 579 | 598 |

^a^ As defined in Figure S1. ^b^*modH* allele the reciprocal exchange was identified in using BLASTn. ^c^ The GenBank accession number for the *modH* allele defined in ^b^. ^c^ The nucleotide number for the 5’ end of the reciprocal exchange. ^d^ The nucleotide number for the 3’ end of the reciprocal exchange
